# Causal associations between fibroblast growth factors and breast cancer: Evidence from 2-sample Mendelian randomization analysis

**DOI:** 10.1097/MD.0000000000043876

**Published:** 2025-08-15

**Authors:** Fubin Feng, Mengxuan Sun, Yan Yao, Huayao Li, Linqi Song, Changgang Sun

**Affiliations:** a Department of Special Medicine, School of Basic Medicine, Qingdao University, Qingdao, China; b Department of Oncology, Weifang Traditional Chinese Hospital, Weifang, China; c First School of Clinical Medicine, Shandong University of Traditional Chinese Medicine, Jinan, China; d College of Traditional Chinese Medicine, Shandong Second Medical University, Weifang, China.

**Keywords:** breast cancer, fibroblast growth factors, mendelian randomization, single-nucleotide polymorphisms

## Abstract

Previous studies have suggested a potential association between fibroblast growth factors (FGFs) and breast cancer (BC), but the evidence for the relationship between specific FGFs with BC is limited and controversial. To explore the interactions between 13 FGFs and 3 fibroblast growth factor receptors (FGFRs) with BC and its subtypes (ER+ and ER−), we conducted a Mendelian randomization (MR) analysis based on genome-wide association study summary statistics of European ancestry. Several techniques were used to ensure the stability of the causal effect, such as inverse-variance weighting, weighted median, MR-Egger regression, and Mendelian Randomization Pleiotropy Residual Sum and Outlier. Heterogeneity was assessed by calculating the Cochran’s *Q* value. The inverse-variance weighting analysis showed that for overall BC, FGF20 showed a genetically protective effect (odds ratio [OR] 0.996, 95% CI: 0.993–1.000, *P = *.027), FGF4 can genetically promote the risk of BC (OR 1.004, 95% CI: 1.001–1.007, *P = *.013). FGF1 (OR 1.055, 95% CI: 1.005–1.107, *P = *.029) and FGF7 (OR 1.068, 95% CI: 1.007–1.133, *P = *.028) were consistently associated with increased risk of ER+ BC, however FGF20 (OR 0.959, 95% CI: 0.920–0.999, *P* = .046) decreased the risk of ER+ BC. FGF23 (OR 1.077, 95% CI: 1.003–1.158, *P = *.042) promote the risk of ER− BC. In the reverse MR study, ER+ BC tended to exhibit elevated levels of FGF7 (OR 1.072, 95% CI: 1.004–1.144, *P* = .037) and decreased levels of FGFR2 (OR 0.930, 95% CI: 0.872–0.992, *P* = .027). Our study results indicate that only specific types of FGFs and FGFRs may have a causal relationship with BC. Th research provides a new perspective on the mechanisms of action of different types of FGFs and FGFRs in BC, and offers potential genetic support for personalized medicine and precision therapy.

## 1. Introduction

Breast cancer (BC) is the most common malignant tumor among woman worldwide, with an estimated 2.3 million new cases and 6,65,684 deaths in 2022, posing a serious threat to women’s health.^[1]^ BC has various pathological types, high incidence, significant tumor heterogeneity, complex mechanisms of occurrence.^[2]^ Traditional clinical subtypes of BC are classified by histological characteristics and the expression of ER, PR, HER2, and Ki-67, resulting in ER-positive, HER2-positive, and triple-negative breast cancer categories. Although these clinical pathological variables are not clear biological parameters, still play a crucial role in assessing prognosis and treatment options.^[3]^ For ER-positive BC, endocrine therapy is standard, controlling tumors by lowering estrogen levels or disrupting ER pathways.^[4,5]^ HER2-positive cancers are treated with targeted therapies like trastuzumab and pertuzumab, improving patient prognosis.^[6,7]^ Comprehensive molecular characterization of triple-negative breast cancer, particularly BRCA mutations and PD-L1 expression profiling, has transformed therapeutic paradigms through precision chemotherapy and immunotherapy. In addition, current clinical trials are evaluating novel targeted therapies directed against TROP-2, EGFR, and the RAS/MAPK signaling pathway.^[8,9]^ Despite the advancements in BC treatment, intrinsic and acquired resistance to targeted drugs and/or classic treatment drugs is a major cause of disease progression, tumor recurrence, and cancer-related deaths. Therefore, understanding the molecular structure and tumor heterogeneity of BC is crucial for effective patient stratification and treatment.^[10]^

In this context, fibroblast growth factors/fibroblast growth factor receptors (FGFs/FGFRs) have attracted widespread attention as an emerging transduction pathway and therapeutic target. The FGFR signaling pathway is activated in various cancers, including BC, lung cancer, gastric cancer, endometrial cancer, and ovarian cancer. In BC, amplification and overexpression of FGFR2 are common genetic alterations associated with tumor aggressiveness and poor prognosis. Additionally, FGFR2 activation of the PI3K-AKT signaling pathway inhibits apoptosis.^[11]^ Dysregulated FGFs/FGFRs signaling may induce oncogenic effects and promote tumor development and metastasis.^[12]^ The FGFs/FGFRs pathway also plays an important role in the tumor microenvironment, affecting the interaction between tumor cells and the surrounding stroma.^[13]^ Studies have shown functional crosstalk between FGFs and hormone receptor signaling pathways, leading to cancer progression and endocrine therapy resistance.^[14,15]^ FGFR1 expression is a recognized poor prognostic factor for ER+ BC.^[16]^ Single-nucleotide polymorphisms (SNPs) of the FGFs/FGFRs pathway also increase the risk of BC and have been widely concerned.^[17]^ A case-control study from the Breast Cancer Association Consortium shows that the FGF10 polymorphism (rs10941679) increases the risk of ER-positive invasive ductal carcinoma.^[18]^ The polymorphisms of FGFR2 (rs1219648, rs2420946, rs2981582) increase the risk of ER and/or progesterone receptor-positive BC in Chinese women.^[19]^ Using the SNaPshot method for genotyping analysis of 747 BC cases and 716 healthy controls, it was found that the genetic variant FGFR1 (rs17182023) was associated with decreased expression levels of the FGFR1 protein and was also related to a reduced risk of BC.^[20]^ Studies have found that there is still considerable controversy regarding the impact of genetic risk variants on FGFRs.^[18,21]^ The causal relationship between FGFs/FGFRs and BC is not well-defined.

Mendelian randomization (MR) uses 1 or more genetic variants as instrumental variables (IVs), which are closely related to the exposure of interest and exclude the influence of confounding factors. The method is widely used as a principal analytical technique to infer the causal relationship between exposure and outcome.^[22]^ Given that ER status is an important biomarker affecting patient prognosis and the selection of treatment strategy, this study employs MR analysis to clarify the causal effects of various FGFs/FGFRs on the risk of BC and its subtypes. Our study may provide a novel perspective for the risk assessment and therapeutic intervention of BC, potentially enhancing our ability to predict and treat this disease more effectively.

## 2. Materials and methods

### 2.1. Study design

**Figure 1.**
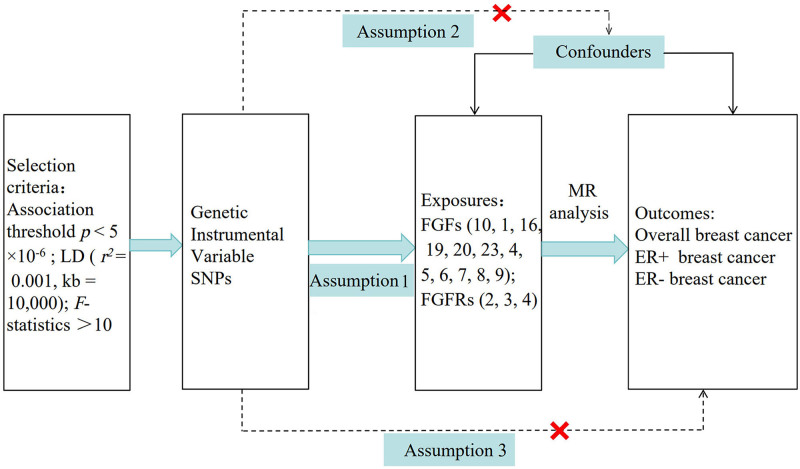
Flow chart of this Mendelian randomization study between FGFs, FGFRs and breast cancer. Assumption 1, denotes the denotes IVs are related with exposure; Assumption 2, the IVs are independent of confounders; Assumption 3, the IVs can only affect outcomes through exposure factors. IVs = instrumental variables, FGFs = fibroblast growth factors, FGFRs = fibroblast growth factor receptors, LD = linkage disequilibrium, SNPs = single-nucleotide polymorphisms, MR = Mendelian randomization.

This research employed a 2-sample MR analysis, carried out to search the reciprocal relationship between 13 types of FGFs (FGF1, FGF4, FGF5, FGF6, FGF7, FGF8 isoform A, FGF8 isoform B, FGF9, FGF10, FGF16, FGF19, FGF20, FGF23) and 3 types of FGFRs (FGFR2, FGFR3, FGFR4) with overall BC and its subtypes (ER+, ER−). Summary statistics from a previously published genome-wide association study (GWAS) was used. Since publicly accessible summary statistics were utilized, no ethical approve was necessary. In our 2-sample MR analysis study, the FGFs/FGFRs and overall BC including its subtypes (ER+, ER−), were considered as exposures and outcomes respectively; whereas in the inverse MR, the exposure and outcome factors were reversed. Genetic variation was employed to evaluate the causal influence exerted by exposure on the outcome. For MR analysis to be valid, adherence to 3 key presuppositions is essential: Relevance: strong correlation between the genetic variants and the exposure; Independence: genetic variants remain unaffected by confounding influences; Exclusion: genetic variants should influence the outcome variables exclusively through the exposure factors^[23]^ (Fig. 1).

### 2.2. Data source

The FGFs, FGFRs were regarded as exposure, and the exposure data were obtained from GWAS summary datasets obtained from the GWAS project (https://gwas.mrcieu.ac.uk/). The BC outcome data were sourced from GWAS datasets. The initial summary statistics for BC were derived from the FinnGen project (https://gwas.mrcieu.ac.uk/).^[24]^ We acquired the GWAS datasets for BC from the Breast Cancer Association Consortium. The compilation encompassed 61,731 affected individuals (38,197 ER+ BC; 9655 ER− BC, 13,879 overall BC) and 2,44,017 unaffected controls.^[17]^ Additional details on the datasets for exposures and outcomes can be found in (Table S1, Supplemental Digital Content, https://links.lww.com/MD/P665).

### 2.3. Selection of genetic Instrumental variables (IVs)

We set the threshold for SNPs related to FGFs, FGFRs at *P*-value <5 × 10^−6^, as potential IVs. We further performed a linkage disequilibrium (LD) analysis for these SNPs (*r*^*2*^ = 0.001, kb = 10,000).^[25]^ The values of *r*^2^ or kb indicate the degree of LD between 2 loci. Beneficial SNPs associated with exposure should not fall within the LD range, as SNPs strongly correlated with LD may lead to biased results. We removed SNPs not found in the GWAS result dataset, as well as palindrome SNPs that could cause bias.^[25]^ SNPs closely related to the outcome (*P* < 5 × 10^−8^) were also removed because they did not meet the key assumptions of IVs. We determined the *f*-statistic for each SNP and omitted those exhibiting low *F*-values (<10) in order to assess potential bias in identifying weak IVs.^[26]^

### 2.4. Statistical analysis

The 2-sample MR were used to explore the causal relationship between FGFs/FGFRs and BC. We employed inverse-variance weighting (IVW), MR-Egger regression, Weighted median, Simple mode, and Weighted mode to ascertain genetic associations. The IVW method is the primary approach in MR analysis due to its superior ability to determine causality. When using the IVW method, the possibility of horizontal pleiotropy must be considered.^[26]^ Cochran’s *Q* test was utilized to assess heterogeneity among IVs with a *P*-value <.05 indicating heterogeneity.^[27]^ If heterogeneity existed, an IVW random effects model was used; otherwise, IVW fixed effects model was used. The MR-Egger method can be used to measure the causal effect.^[27]^ Horizontal pleiotropy is assessed by calculating the intercept of the MR-Egger regression and Mendelian Randomization Pleiotropy Residual Sum and Outlier (MR-PRESSO). The leave-one-out method determines whether the results are significantly affected after removing a single SNP.^[28]^ We use the odds ratio (OR) to express the impact of exposure factors on the risk of outcomes. Consistency across the 5 methods, particularly with a significant finding from the IVW method, was considered indicative of a reliable association.^[29]^ The statistical analysis was conducted using R software (version 4.2.0), with the R packages “two-sample MR” and “MR-PRESSO.” In this study, statistical significance threshold was set at the level of *P* < .05.

### 2.5. Reverse MR analysis

To explore the causal effect of overall BC and its subtypes (ER+ and ER−) on specific FGFs and FGFRs, we performed a reverse MR analysis, considering BC as the exposure and the FGFs, FGFRs as the outcomes.

## 3. Results

### 3.1. Causal associations between FGFs, FGFRs and breast cancer

**Figure 2.**
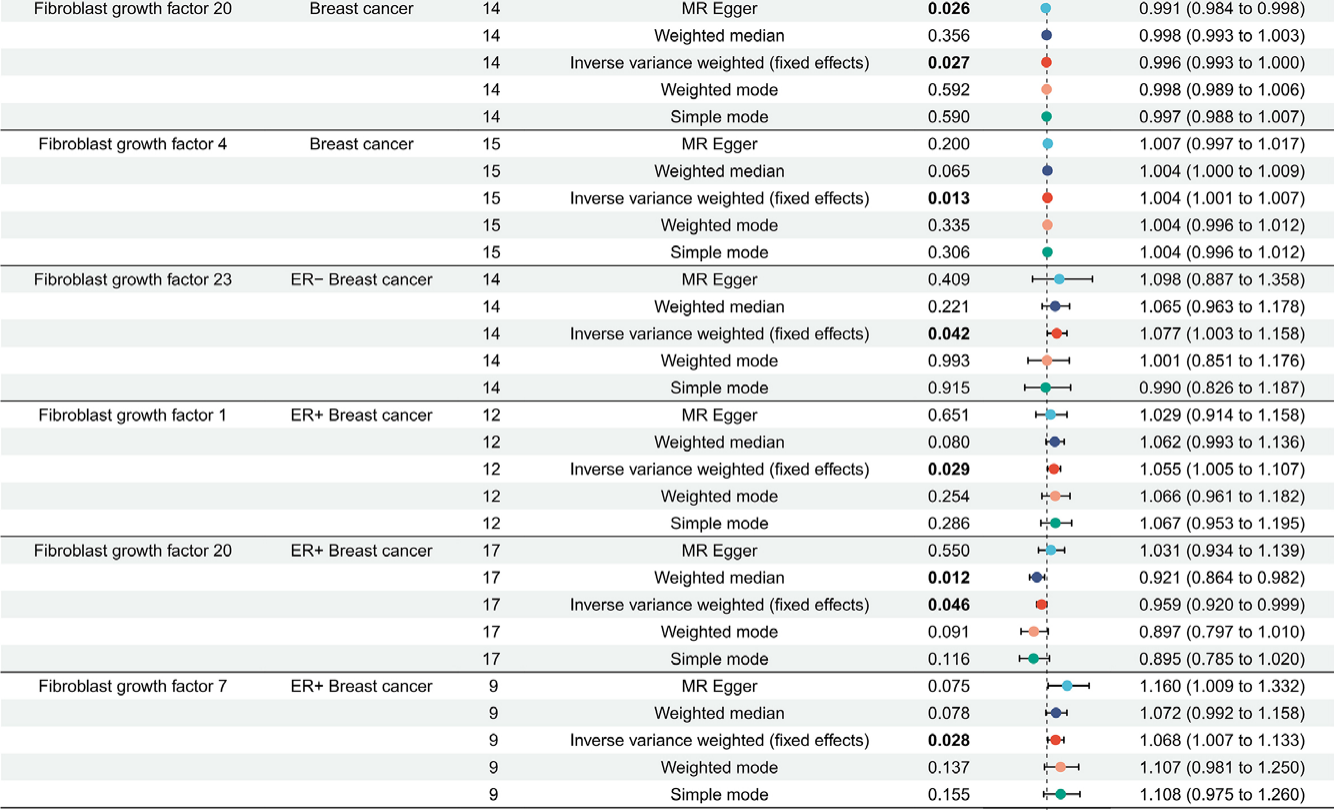
Forest plots showed the results of FGFs, FGFRs on risk of breast cancer based on the IVW model. Five MR methods were employed to ascertain the relationship between selected exposure (FGF20, FGF4, FGF23, FGF1, FGF7) on risk of breast cancer. The IVW method was considered the primary method depicted in this illustration. Causal estimates express the change in odds ratio (OR), Error bars indicate 95% confidence intervals. FGFs = fibroblast growth factors, FGFRs = fibroblast growth factor receptors, IVW = inverse-variance weighting, MR = Mendelian randomization, OR = odds ratio.

**Figure 3.**
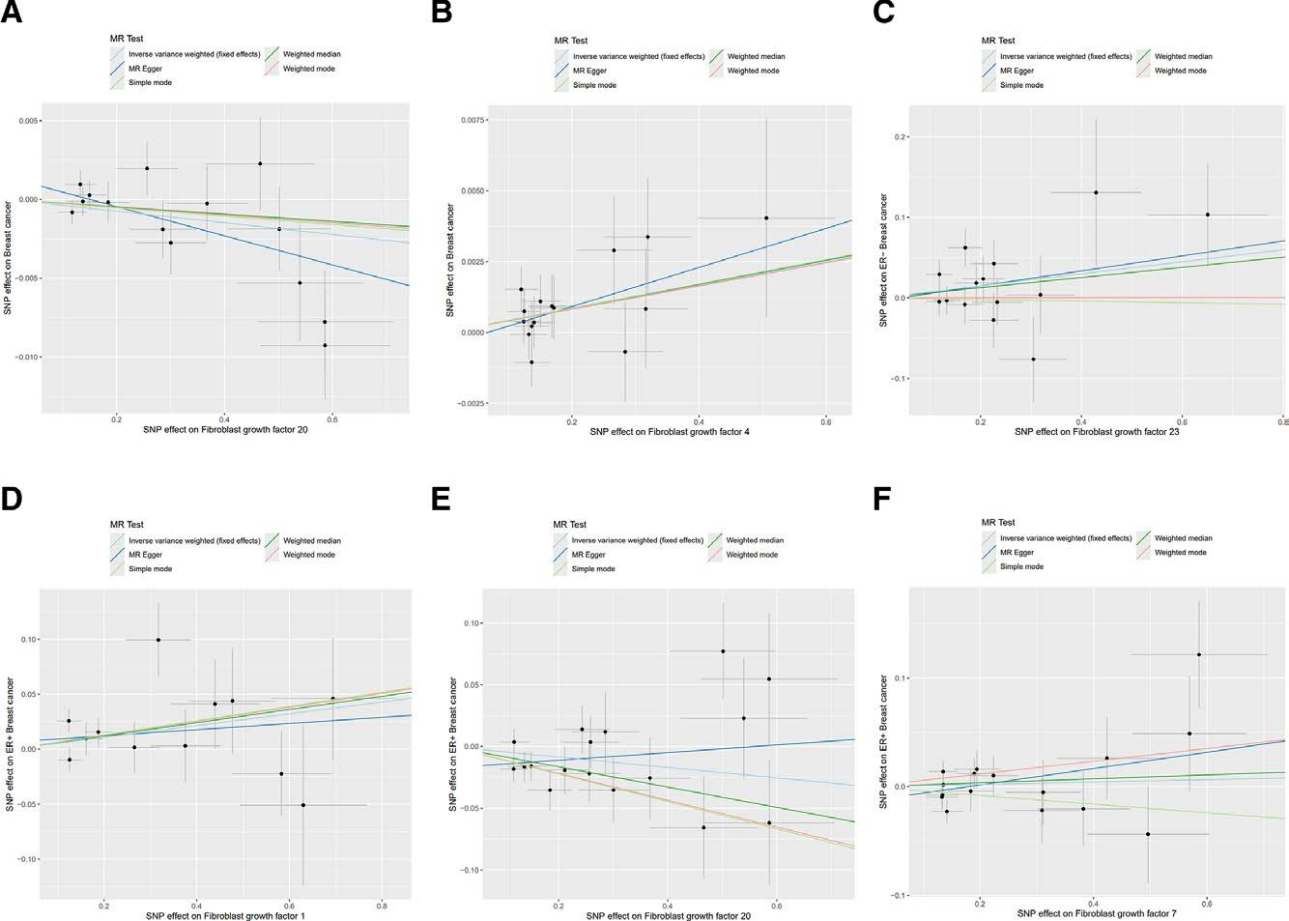
Scatter plot illustrating the genetic correlations between FGFs, FGFRs and breast cancer. The horizontal axis of the scatter diagrams represents the causal impact of instrumental variables (IVs) on the outcome, while the vertical axis indicates the causal impact of the IVs on the exposure. The gradients of the lines indicate the causal effect attributed to each method. (A) Scatter diagram of genetic correlations for levels of FGF20 in relation to overall breast cancer. (B) FGF4 in relation to overall breast cancer. (C) FGF23 in relation to ER− breast cancer. (D) FGF1 in relation to ER+ breast cancer. (E) FGF20 in relation to ER+ breast cancer. (F) FGF7 in relation to ER+ breast cancer. IVs = instrumental variables, FGFs = fibroblast growth factors, FGFRs = fibroblast growth factor receptors,

In the present study, we explored the causal relationships between FGFs, FGFRs, and BC, including its subtypes ER+ and ER− BC. Applying rigorous criteria significance threshold (*P* < 5 × 10^−6^), and LD (*r*^*2*^ = 0.001, kb = 10,000), 4–16 SNPs were left as IVs for FGFs, FGFRs. The *F*-statistics for the genetic instruments ranged from 20.8 to 64.3, indicating these SNPs are robust and our study was not likely to be affected by weak instrument bias (Table S1, Supplemental Digital Content, https://links.lww.com/MD/P665). Utilizing these SNPs, we conducted MR analysis using various methods. Our findings indicate a genetically protective effect of FGF20 on overall BC (OR 0.996, 95% CI: 0.993–1.000, *P = *.027), while FGF4 was associated with an increased risk of overall BC (OR 1.004, 95% CI: 1.001–1.007, *P* = .013). For ER+ BC cases, FGF1 (OR 1.055, 95% CI: 1.005–1.107, *P* = .029) and FGF7 (OR 1.068, 95% CI: 1.007–1.133, *P* = .028) were consistently linked to a higher risk of BC. Conversely, FGF20 (OR 0.959, 95% CI: 0.920–0.999, *P* = .046) was found to reduce the risk of BC, with consistent results from the weighted median (OR 0.921, 95% CI: 0.864–0.982, *P* = .012) and MR-Egger (*P* = .550). For ER-BC FGF23 (OR 1.077, 95% CI: 1.003–1.158, *P* = .042) promote the risk of BC (Fig. 2, Table S2, Supplemental Digital Content, https://links.lww.com/MD/P666). No significant heterogeneity was detected by Cochran’s *Q*-test or the MR-Egger intercept analysis (Table S3, Supplemental Digital Content, https://links.lww.com/MD/P667). What needs to be primarily noted is, OR values (such as FGF20 for ER+ BC) are <1 but very close to 1, with statistical difference in *P* value; combined with the results of weighted median and MR-Egger analysis, we further believe that these exposure factors have a protective effect on BC. We found that there was no clear causal relationship between FGFRs (2, 3, 4) and overall BC, with similar results observed across subtypes (Table S2, Supplemental Digital Content, https://links.lww.com/MD/P666). The available data provided scant evidence for a causal link between the remaining FGFs, FGFRs and the risk of BC. The scatter diagrams illustrated the distinct impacts of each approach on individual outcomes in the databases (Fig. 3).

### 3.2. Sensitivity and heterogeneity analysis

**Figure 4.**
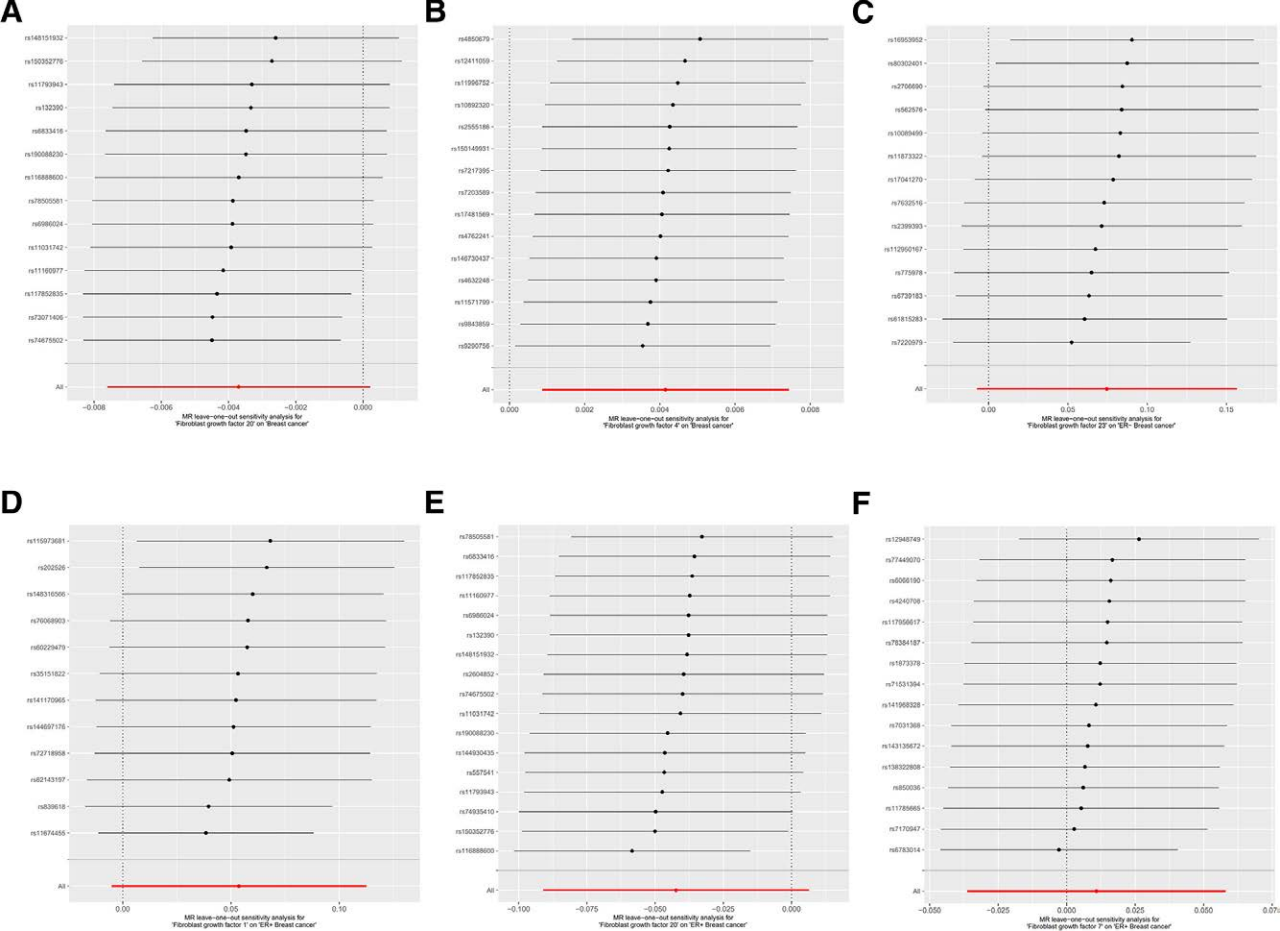
Leave-one-out sensitivity 2-sample MR analysis based on the IVW model for FGFs on risk of breast cancer. (A) FGF20 on overall breast cancer, (B) FGF4 on overall breast cancer, (C) FGF23 on ER− breast cancer, (D) FGF1 on ER+ breast cancer, (E) FGF20 on ER+ breast cancer, (F) FGF7 on ER+ breast cancer. FGFs = fibroblast growth factors, IVW = inverse-variance weighting, MR = Mendelian randomization.

To ensure the robustness of the causal estimates, we conducted sensitivity and heterogeneity analyses for each MR analysis. The Cochran *Q* test was used to assess heterogeneity, revealing no significant variability in the correlations for exposure factors with positive results (*P*-value >.05). The Egger intercept test showed no evidence of horizontal pleiotropy (*P*-value >.05), and the funnel plots displayed a symmetrical SNP distribution (Fig. S1, Supplemental Digital Content, https://links.lww.com/MD/P664). Additionally, MR-PRESSO analysis confirmed the absence of significant horizontal pleiotropy in the positive results (Table S3, Supplemental Digital Content, https://links.lww.com/MD/P667). Furthermore, the leave-one-out (Fig. 4), forest plots (Fig. S2, Supplemental Digital Content, https://links.lww.com/MD/P664) for analysis provided additional confirmation for the causal estimates of FGFs and FGFRs.

### 3.3. Reverse MR analysis for causal effect of breast cancer on FGFs and FGFRs

**Figure 5.**
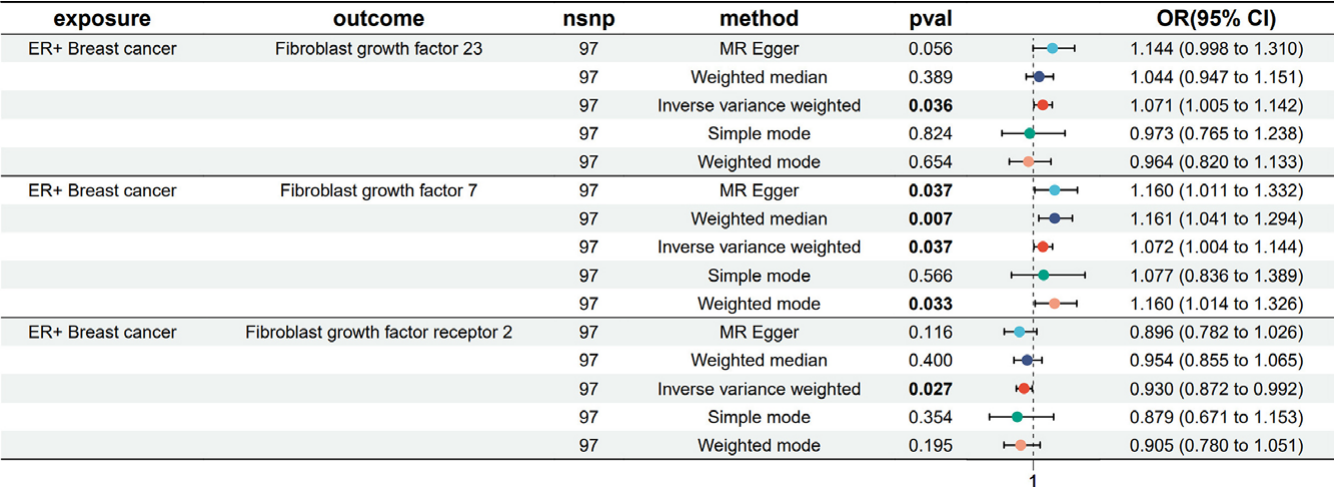
Forest plots showed the causal associations between breast cancer and FGFs, FGFRs by using different methods in reverse MR analysis. The IVW method was considered the primary method depicted in this illustration. Causal estimates express the change in odds ratio (OR), Error bars indicate 95% confidence intervals. CI = confidence intervals, FGFs = fibroblast growth factors, FGFRs = fibroblast growth factor receptors, IVW = inverse-variance weighting, MR = Mendelian randomization, OR = odds ratio.

**Figure 6.**
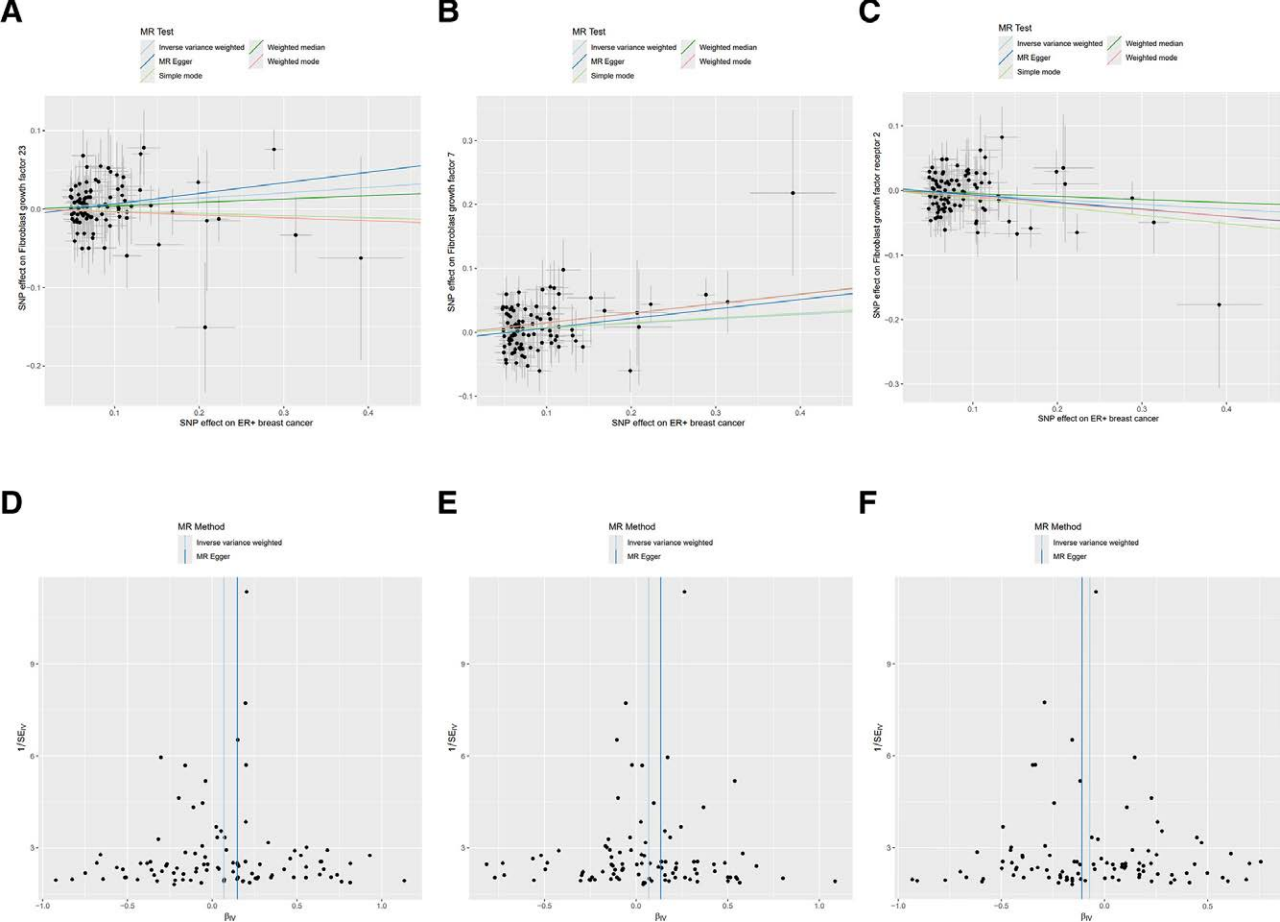
Scatter and funnel plots of reverse MR analyses for breast cancer on FGFs and FGFRs levels. The gradients of the lines indicate the causal effect attributed to each method. Scatter diagram of genetic correlations for ER+ breast cancer in relation to (A) FGF23, (B) FGF7, and (C) FGFR2 respectively. The funnel plots show the IVW-MR estimate of each (D) FGF23, (E) FGF7, and (F) FGFR2 SNP with breast cancer versus 1/standard error (1/SEIV). FGFs = fibroblast growth factors, FGFRs = fibroblast growth factor receptors, IVW = inverse-variance weighting, MR = Mendelian randomization, OR = odds ratio, SNP = single-nucleotide polymorphism.

To further explore the causal relationship between BC and FGFs/FGFRs, we conducted a reverse MR analysis, defining all BC, including ER− and ER+ BC as exposures and FGFs, FGFRs as outcomes. Utilizing independent SNPs as IVs, our IVW-MR analysis revealed that individuals genetically predisposed to ER+ BC are likely to have increased levels of FGF7 (OR 1.072, 95% CI: 1.004–1.144, *P* = .037) and FGF23 (OR 1.071, 95% CI: 1.005–1.142, *P* = .036), and decreased levels of FGFR2 (OR 0.930, 95% CI: 0.872–0.992, *P* = .027; Fig. 5). Table S4, Supplemental Digital Content, https://links.lww.com/MD/P668 provides an extensive overview of the reverse MR analysis using 5 methodologies. Heterogeneity was not detected, as evidenced by the Cochran’s *Q*-test and the MR-Egger intercept analysis, detailed in Table S5, Supplemental Digital Content, https://links.lww.com/MD/P669. Scatter and funnel plots representation of MR analyses for FGFs and FGFRs in breast were exhibited in Figure 6.

## 4. Discussion

Although 2-sample MR analysis has been widely used in similar studies, this study represents our first application of this method to investigate the specific relationships between FGFs, FGFRs, and BC risk. Employing multiple methodologies, we validated the stability of the causal effects. Our 2-sample MR analysis indicates causal associations between certain FGFs and BC: FGF4 as a potential risk factor, and FGF20 as a protective factor for overall BC. The causal effects of FGFs on BC subtypes show specificity, FGF20 potentially serves as a protective factor for ER+ BC, without a similar effect for ER-subtype. In contrast, FGF23 is suggested as a risk factor for ER− BC, with no such association observed for ER+ BC.

Our MR study identified FGF4 as a potential risk factor for overall BC (OR 1.004, 95% CI: 1.001–1.007, *P* = .013). Recognized as a pleiotropic growth factor, the role of FGF4 in cancer progression is well-documented.^[30,31]^ Specifically in BC, it is linked to increased tumor growth, progression, and therapeutic resistance.^[32,33]^ Shi Hui’s research indicated that FGF4 is overexpressed in the metastatic lymph nodes of BC patients, and the oncoprotein HBXIP promotes its expression through Sp1, enhancing cancer cell migration.^[34]^ MiR-511, acting as a regulator of FGF4, can suppress BC cell proliferation and metastasis by reducing FGF4 levels.^[35]^ Collectively, these findings indicate FGF4 may play a pivotal role in the progression and metastasis of BC. The oncogenic effect is mainly manifested in overall BC, not restricted by ER status, which confirms the reliability of our results.

For ER+ BC, the main risk factors identified are FGF1 (OR 1.055, 95% CI: 1.005–1.107, *P* = .029) and FGF7 (OR 1.068, 95% CI: 1.007–1.133, *P* = .028); no causal relationships were observed in ER− BC. In the reverse MR analysis, individuals of ER+ BC exhibited a positive causal relationship with FGF7 (OR 1.072, 95% CI: 1.004–1.144, *P* = .037). FGF1 is closely associated with endocrine resistance, with most research focusing on ER+ BC. FGF1 binds to FGFR1 and activates ERαin BC cells, potentially stimulating cancer progression even after estrogen deprivation.^[36]^ FGF1 activates the MAPK and PI3K signaling pathways through FGFR3, leading to tamoxifen resistance in MCF-7 cells.^[37]^ Additionally, when MCF-7 cells transfected with FGF1 are transplanted into ovariectomized mice, they promote the occurrence of endocrine-resistant tumors.^[38]^ FGF7 is another significant risk factor for the development of ER+ BC. Distinguished from FGF1, FGF7 may primarily act in synergy with progesterone to escalate tumor development and metastasis. Piasecka D found that FGF7 can enhance the growth and migration of PR-dependent MCF7 cells, downregulate PR through the RSK2-mediated pathway, contributing to the development of steroid hormone-negative BC.^[39]^ The activation of the FGF7/FGFR2 axis can independently regulate the phosphorylation of ER and PR, leading to tamoxifen resistance in BC.^[14]^ This axis, in conjunction with Jun-B can eliminate the regulatory effect of PR on ER-related biological functions in premenopausal ER+ BC, promoting the occurrence and progression of tumors.^[40]^ This suggests that FGF7 may be a risk factor for ER+ BC, affecting the expression of PR expression, and modulate tumor biology and response to hormonal therapies.

Our research suggested that FGF23 as a potential risk factor uniquely associated with ER-negative BC (OR 1.077, 95% CI: 1.003–1.158, *P* = .042). Despite its limited study in BC biology, FGF23 is notably implicated in the biological mechanisms of bone metastasis in cancer.^[41]^ Although 3 SNPs of FGF23 are associated with an elevated risk of prostate cancer,^[42]^ its causal connections to other cancers are less documented. Higher FGF23 serum levels correlate with reduced survival in patients with tumors and bone metastases,^[43]^ and there is upregulation of FGF23 mRNA in BC.^[44]^ While current research predominantly investigates the correlation between FGF23 and BC, it does not delve into causality. Considering FGF23’s role in bone metastasis and its unique link to ER- BC, there is a critical need for further studies to clarify its regulatory effects on the occurrence of bone metastasis in BCs with different hormone receptor statuses.

In the overall BC population and the ER+ subgroup, FGF20 correlates with a lower risk of developing the disease, with ORs of 0.996 (95% CI: 0.993–1.000, *P = *.027) and 0.959 (95% CI: 0.920–0.999, *P* = .046), respectively. This positions FGF20 as a unique protective element among FGFs. Known as a neurotrophic factor, FGF20 is involved in nervous system development and is prevalent in glioma cells.^[45]^ In the nervous system, FGF20 has been proven to protect dopaminergic neurons. For example, studies have found that FGF20 can reduce the loss of dopaminergic neurons in vitro and provide functional protection in a rat model of Parkinson’s disease induced by 6-hydroxydopamine.^[46]^ In addition, FGF20 promotes angiogenesis and vascular repair after traumatic brain injury by regulating the Wnt/β-catenin signaling pathway.^[47]^The expression level of FGF20 in BC is relatively low. Compared with other members of the FGF family, such as FGF9, its expression in BC is not significant.^[48]^ Research has found that FGF20 is expressed in embryonic mammary glands and is an important mediator in the morphogenesis of mammary gland budding and branching.^[49]^ The exact molecular mechanisms of FGF20 influence on mammary morphogenesis are yet to be fully elucidated. Further research is warranted to determine if FGF20’s protective effect in BC is mediated through developmental processes. While FGF10, another cytokine with established in vivo roles in mammary gland development,^[50]^ impacts early stages of mammary organogenesis. Our MR analysis in the ER+ group did not reveal a significant correlation with BC risk (OR 1.017, 95% CI: 0.951–1.088, *P* = .619). The protective role of FGF20 in BC merits critical appraisal, with future studies required to delineate its specific functions and therapeutic implications. We did not identify a causal link between FGFR receptors (FGFR2, 3, 4) and BC. However, in a reverse MR analysis, a genetic predisposition for ER+ BC was associated with reduced FGFR2 levels (OR 0.930, 95% CI: 0.872–0.992, *P* = .027). FGFR2 signaling is known to antagonize estrogen signaling pathways, potentially increasing the risk of ER+ BC through its influence on estrogen response.^[51]^ Additionally, the role of FGFR2 varies throughout the stages of mammary gland development, and there is ongoing debate regarding the impact of genetic risk variants on FGFR expression.^[21,52]^

Both in vitro and in vivo studies have demonstrated that abnormal FGF/FGFR signaling may be associated with the occurrence and development of breast tumors.^[53]^ Moreover, overexpression and/or abnormal activation of FGFRs are often related to acquired resistance to established cancer therapies.^[54]^ A variety of therapeutic approaches targeting the FGF/FGFR signaling pathway have recently been developed.^[55,56]^ Lenvatinib (E7080), a small molecule targeting FGFR1-4, VEGFR1, PDGFR, RET, and KIT, has been shown to inhibit lymph node and lung metastasis in BC xenograft models.^[57]^

Our findings are robust, having thoroughly examined the causal links between various FGFs and FGFRs in relation to BC, unaffected by horizontal pleiotropy and other potential confounding factors, ensuring the reliability and validity of the study outcomes. Secondly, the SNPs used as IVs are closely related to BC, excluding potential weak instrument bias. Additionally, the MR-PRESSO method was employed to identify and correct biases caused by outliers. However, our study has limitations. Firstly, we did not investigate the correlation between FGFs, FGFRs and other types of cancer. Secondly, due to the types of data provided by public databases, we did not study all types of FGFs and FGFRs but selected representative indicators with complete data as exposure factors, which has certain limitations. Furthermore, most of our data pertains to individuals of European descent, and whether our study results can be generalized to other ethnicities requires further research.

## 5. Conclusion

In this comprehensive MR study, we used GWAS summary statistics from individuals of European descent to explore the causal relationship between FGFs/FGFRs and BC. The findings indicate that only some specific FGFs may have a causal relationship with BC. Simultaneously, the genetic liability to BC risk may has a causal effect on the levels of specific FGFs and FGFRs. The study offers new insights into the mechanisms of action of different types of FGFs and FGFRs in breast cancer and provides potential genetic support for personalized medicine and precision therapy.

## Acknowledgments

The authors express gratitude to the Cancer Research Alliance for providing researchers with high-quality GWAS summary statistics. Thanks to the participants and researchers of the FinnGen study.

## Author contributions

**Conceptualization:** Changgang Sun.

**Data curation:** Fubin Feng.

**Formal analysis:** Linqi Song.

**Funding acquisition:** Changgang Sun.

**Methodology:** Mengxuan Sun, Linqi Song.

**Resources:** Changgang Sun.

**Software:** Yan Yao, Huayao Li.

**Supervision:** Yan Yao, Changgang Sun.

**Visualization:** Mengxuan Sun.

**Validation:** Huayao Li.

**Writing** – **original draft:** Fubin Feng.

**Writing** – **review & editing:** Fubin Feng, Huayao Li, Changgang Sun.

## Supplementary Material


