# Supplementary material for: Causal associations between fibroblast growth factors and breast cancer: Evidence from 2-sample Mendelian randomization analysis
Source: Medicine (Baltimore). 2025 Aug 15;104(33):e43876. doi: 10.1097/MD.0000000000043876 (PMC12366922; doi:10.1097/MD.0000000000043876)

**Figure S1**

Funnel plots illustrate the causal relationships between FGFs, FGFRs and breast cancer in MR analyses. These plots depict the influence of SNPs on the connection between these FGFs, FGFRs and breast cancer. (A) Funnel plot of the causal effects of FGF20 on overall breast cancer, (B) FGF4 on overall breast cancer, (C) FGF23 on ER- breast cancer, (D) FGF1 on ER+ breast cancer, (E) FGF20 on ER+ breast cancer, (F) FGF7 on ER+ breast cancer.


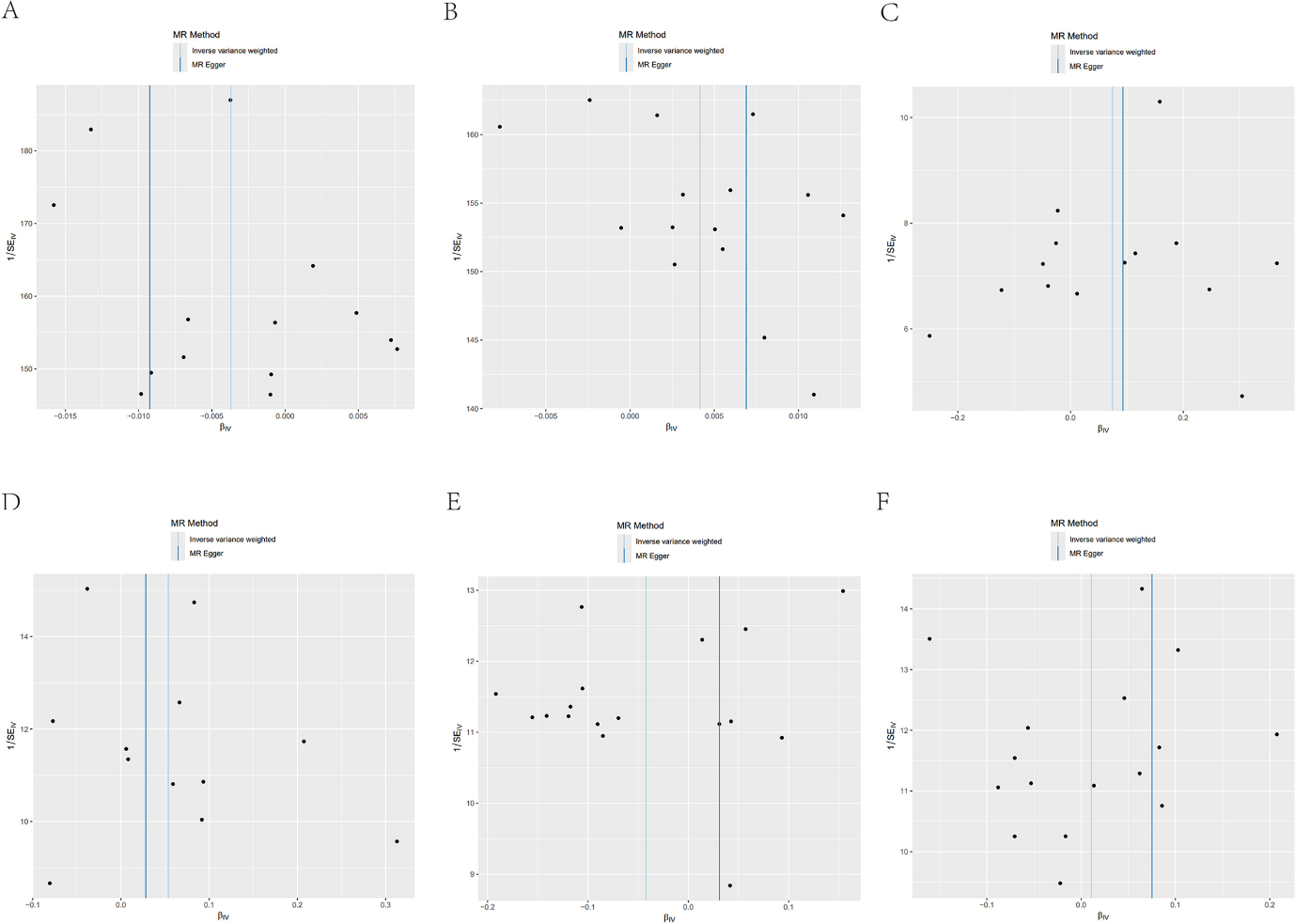


**Figure S2**

Forest plots for MR analyses of the causal effect of FGFs, FGFRs on breast cancer. The red and black dots/bars within these forest plots signify the causal estimates of the effect of FGFs, FGFRs on breast cancer. (A) Causal effects of FGF20 levels on overall breast cancer, (B) FGF4 levels on overall breast cancer, (C) FGF23 levels on ER- breast cancer, (D) FGF1 levels on ER+ breast cancer, (E) FGF20 levels on ER+ breast cancer, (F) FGF7 levels on ER+ breast cancer.


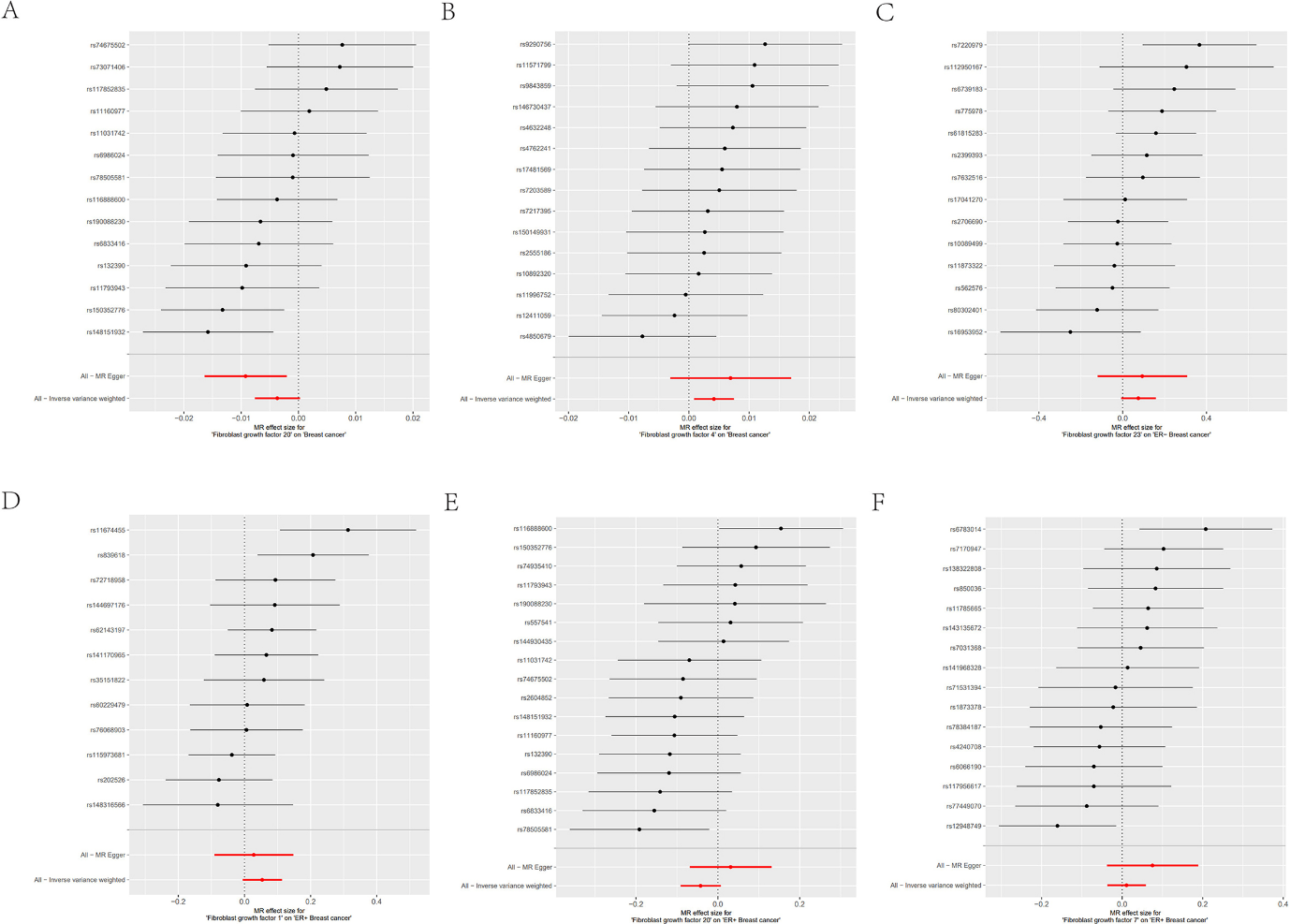

Supplement: Supplementary file 4 [file medi-104-e43876-s004.docx]
